# The Association between Metals and Thyroid Cancer in Puerto Rico—A National Health and Nutrition Examination Survey Analysis and Ecological Study

**DOI:** 10.3390/toxics12090632

**Published:** 2024-08-28

**Authors:** Yaelle Shaked, Jessica Yang, Mathilda Monaghan, Maaike van Gerwen

**Affiliations:** 1 Department of Otolaryngology-Head and Neck Surgery, Icahn School of Medicine at Mount Sinai, New York, NY 10029, USA; yaelle.shaked@icahn.mssm.edu (Y.S.); jessica.yang@icahn.mssm.edu (J.Y.); mathilda.monaghan@mountsinai.org (M.M.); 2 Institute for Translational Epidemiology, Icahn School of Medicine at Mount Sinai, New York, NY 10029, USA

**Keywords:** environmental exposures, thyroid cancer, lead, mercury, cadmium

## Abstract

Thyroid cancer rates have risen globally over the past four decades, with Puerto Rico experiencing a particularly pronounced increase. This may be linked to higher metal exposure, as some metals are endocrine disruptors and carcinogens. Currently, certain regions of Puerto Rico have Superfund programs because of high concentrations of metals in the soil. Therefore, we investigated the association between thyroid cancer incidence and three metals (lead, cadmium, and mercury) with known endocrine-disrupting properties and increased levels in soil samples in Puerto Rico. We used the National Health and Nutrition Examination Survey (NHANES) data for heavy metal levels and the thyroglobulin antibody (TgAb) as a thyroid cancer marker. Additionally, we performed an ecological study using data from the Environmental Protection Agency (EPA) report on Metals from Natural and Anthropogenic Sources in Puerto Rico Soils and data from the Puerto Rico Central Cancer Registry on age-adjusted thyroid cancer incidence rates from 2015 to 2019. Through NHANES analysis, we found a significant negative association between mercury and TgAb. Through our ecological study, we observed higher thyroid cancer incidence rates and increased metal levels in the soil in the northern parts of Puerto Rico. Our heterogenous results necessitate further research on this topic.

## 1. Introduction

Global thyroid cancer incidence rates have increased steadily over the past four decades [1,2]. However, the epidemiological landscape of thyroid cancer incidence is not evenly distributed [3]. Puerto Rico is one region that has seen an inordinate number of thyroid cancer cases. According to the Global Cancer Observatory (2018), Puerto Rico has the highest incidence rate of cancer in the Americas and the fourth highest rate worldwide [4]. From 2016 to 2020, Puerto Rico’s age-adjusted thyroid cancer incidence rate (25.6 cases/100,000 people) was twice that of the United States (13.3 cases/100,000 people) [5].

The increasing thyroid cancer incidence rates have been attributed to different causes, including access to diagnostic modalities, leading to potential overdiagnosis [6]. On the other hand, additional factors to be considered include exposure to ionizing radiation, obesity, and environmental pollutants, like pesticides and industrial chemicals [7,8,9,10]. Location-specific exposure is also important to explore as higher thyroid cancer incidence rates have been found in specific areas, such as volcanic regions. Studies in Hawaii, Iceland, and the French Polynesian islands, all islands with previous or current robust volcanic activity, have noted very high thyroid cancer incidence rates [11,12,13,14]. An epidemiological study of thyroid cancer incidence in Sicily, a Mediterranean island home to the active volcano Mount Etna, further illustrated the association between volcanic activity and thyroid cancer. In a comparison of two populations with similar lifestyles, genetic, and ethnic compositions in two adjacent areas on the island from 2002 to 2004, it was found that the population directly adjacent to the volcano had approximately double the risk of papillary thyroid cancer. The incidence rates were 31.7 cases/100,000 residents (females) and 6.4 cases/100,000 residents (males) compared to 14.1 cases/100,00 residents (females) and 3.0 cases/100,000 residents (males) in the population not directly adjacent to the volcano [15]. Further investigating thyroid cancer in Puerto Rico is of interest because it was formed by a once-active volcano [16].

Heavy metals found in soil and water in volcanic regions have been hypothesized to be one of the reasons for increased thyroid cancer incidence rates in populations near these geographical landmarks [11]. Although heavy metals are a natural part of the Earth’s crust, heavy metals such as mercury (Hg), lead (Pb), and cadmium (Cd) are released into the surrounding environment when a volcano erupts, leading to higher soil concentrations in adjacent areas [17]. As a result, surrounding organisms take in these heavy metals either through direct contact or ingestion. In a follow-up study, elevated levels of Hg, Pb, and Cd were found in both tap water and lichens from the area around Mount Etna in comparison to a control area [18]. High levels of Hg, Pb, and Cd also translate to elevated heavy metal blood levels in the surrounding population [19,20,21].

Besides its volcanic origin, Puerto Rico exhibits significant levels of soil contamination, which can be observed by the high number of Superfund sites spread across the island. As of January 2024, the island has 16 active Superfund sites after recently closing three sites because of cleanup. Superfunds are programs funded by the United States (US) Environmental Protection Agency (EPA) and devoted to cleaning up contaminated sites where hazardous waste has been dumped, left in the open, or otherwise improperly managed [22]. Hg, Pb, and Cd are three heavy metals with identified increased concentrations in Puerto Rican soil [23]. Although certain heavy metals play an important role in the function of the thyroid gland, some metal ions are known to disrupt endocrine function (e.g., Cd, Pb, Hg) or may even be carcinogenic (e.g., Cd), which may explain their association with thyroid cancer [9,24,25].

Building on the existing literature on the association between heavy metal exposure and thyroid cancer, particularly in volcanic regions, we performed two separate analyses: (1) an assessment of the association between identified increased heavy metal ions in volcanic soil (Hg, Pb, and Cd) and thyroglobulin antibodies (TgAb) as potential markers of thyroid cancer, using the National Health and Nutrition Examination Survey (NHANES), and (2) an ecological study of Hg, Pb, and Cd levels and thyroid cancer incidence rates per county in Puerto Rico, as an area with known high levels of these heavy metals due to volcanic activity and the presence of Superfund sites as well as high thyroid cancer incidence rates.

## 2. Materials and Methods

### 2.1. NHANES Analysis

#### 2.1.1. Data Source and Study Population

The NHANES is a cross-sectional survey of the health and nutrition status of the civilian, non-institutionalized population in the US, administered by the National Center for Health Statistics. The NHANES uses a complex, multistage, probability sampling design that selects representative groups of the population and has released data in two-year cycles since 1999. The NHANES program was approved by the National Center for Health Statistics (NCHS) Ethics Review Board (protocol #2005-06 and protocol #2011-17 (effective through 26 October 2017), covering the included study years (see below)).

For this study, we used TgAb as a primary outcome measure, and anti-thyroid-peroxidase (anti-TPO), as a secondary outcome measure. TgAb was first discovered in patients with autoimmune thyroid disease [26]. Clinically, the presence of TPO suggests the presence of an autoimmune disorder [27]. Anti-TPO is more common than TgAb and may be more indicative of a thyroid disease [28]. Positive serum TgAb was found to be more than twice as high in patients with differentiated thyroid cancers (DTCs) than in the general population. In the same study, the presence of either TgAb or anti-TPO was found to be nearly three times greater in patients with DTCs than the general population [29]. Furthermore, in a retrospective review, patients with a thyroid nodule and preoperative TgAb ≥ 30 IU/mL had a higher rate of malignancy when compared to patients with TgAb  <  30 IU/mL [30]. Therefore, TgAb is of specific interest as a primary outcome measure to serve as a proxy for thyroid cancer.

**Figure 1:**
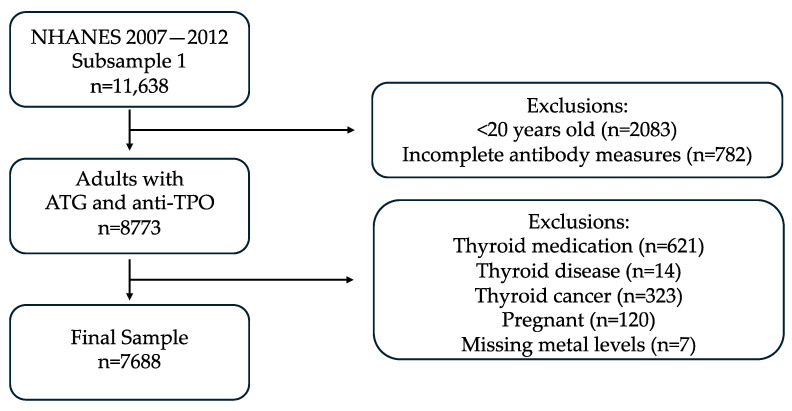
Selection criteria.

For this study, we selected participants from 2007 to 2012 who were >20 years old and had complete TgAb and anti-TPO laboratory measures (n = 8773). We then excluded participants who were currently on thyroid medication as defined by Christensen et al. and Turyk et al. (n = 621), had thyroid disease (n = 14), had thyroid cancer (n = 323), and were pregnant (n = 120) [31,32]. Participants with missing metal levels were also excluded (n = 7), which resulted in a final sample size of 7688 patients (Figure 1).

#### 2.1.2. Outcomes and Covariates

The primary outcome was TgAb, and the secondary outcome was anti-TPO. NHANES laboratory documentation was used to obtain reference ranges for TgAb and anti-TPO levels; normal ranges for TgAb and anti-TPO were <4 IU/mL and <9 IU/mL, respectively [33]. Detailed information on specimen collection and processing can be found in the NHANES Laboratory Procedures Manual [34]. Our primary predictors were Pb, Cd, and Hg. Pb, Cd, and Hg were determined in whole blood using inductively coupled plasma mass spectrometry. A detailed description of the laboratory methods can be found in the NHANES 2007–2008, 2009–2010, and 2011–2012 Lab Methods for Cadmium, Lead, Total Mercury, Selenium, and Manganese—Blood [35,36,37]. Values below the limit of detection for each metal were imputed as lower limits of detection (LLOD)/√2, which is consistent with NHANES practices. Covariates of interest included age, sex, race/ethnicity, body mass index (BMI), iodine status, and smoking status. Age was categorized into groups (<40 years, 40–59 years, ≥60 years). BMI was categorized following cut-offs proposed by the Center for Disease Control and Prevention (CDC): <25 kg/m^2^, 25–30 kg/m^2^, and >30 kg/m^2^ [38]. Iodine status was defined as normal (≥100 µg/L urine) and low (<100 µg/L urine), following the recommendations of the World Health Organization [39]. Smoking status was defined using serum cotinine, a metabolite of nicotine. Cotinine is used as a biomarker to distinguish smokers from nonsmokers in epidemiological studies. Serum cotinine levels were used to classify smokers and nonsmokers using cut-off points based on race/ethnicity, as described by Benowitz et al. [40].

#### 2.1.3. Statistical Analysis

To account for the complex, multi-stage, probability sampling strategy used by NHANES and to produce nationally representative estimates, we used survey procedures and incorporated survey design variables and weights. All results shown represent weighted values.

Thyroid antibodies, Pb, Cd, and Hg were natural log (ln)-transformed. The association between the different metals and the two thyroid antibodies was examined using multivariable regression models (PROC SURVEYREG) with the metals as both a continuous predictor and divided into quartiles, similar to the approach by Christensen et al. [31]. All models were adjusted for sex, age, race/ethnicity, BMI, iodine status, and smoking status. The analyses were performed using SAS software, version 9.4 (SAS Institute Inc., Cary, NC, USA).

### 2.2. Ecological Study

#### 2.2.1. Thyroid Cancer Incidence

County-level age-adjusted thyroid cancer incidence rates (per 100,000 people) were taken from the Puerto Rico Central Cancer Registry (PRCCR), a population registry established by the Department of Health in 1950. It collects information on cancer cases diagnosed among residents of Puerto Rico. For this study, cancer data from 2015 to 2019 were used [41]. These data were imported into ArcGIS to display age-adjusted incidence rates of thyroid cancer per county. ArcGIS is a mapping software developed by the Environmental Systems Research Institute (version 10:8; ESRI, Redlands, CA, USA).

#### 2.2.2. Heavy Metal Measurements

The locations of Pb, Cd, and Hg were taken from the Environmental Protection Agency (EPA) report: Metals from Natural and Anthropogenic Sources in Puerto Rico Soils [23]. Metal levels were reported in parts per million (ppm). The soil samples were collected in the years 1989, 1990, 1992, 1994, 1997, 1998, 1999, 2000, 2004, 2005, 2006, 2007, 2008, 2011, and 2013. During this time, there were 19 Superfund sites in Puerto Rico.

#### 2.2.3. Statistical Analysis

The locations of Pb, Cd, and Hg with a soil concentration of 400, 0.48, and 0.81 ppm, respectively, were overlaid on the map of age-adjusted thyroid cancer incidence rates per county using ArcGIS. These levels of concentration for each heavy metal are above the EPA’s maximum contaminant level (MCL) for residential soil. Due to the missing data of metal locations for a large number of counties, no correlation analysis was performed and data were only visually interpreted [23].

## 3. Results

### 3.1. NHANES Analysis

Of the 7688 participants, most participants were male (52.9%), non-Hispanic White (67.8%), and between 40 and 59 years (39.4%). About one-third of participants had a BMI < 25 kg/m^2^, one-third had a BMI between 25 and 30 kg/m^2^ (34.1%), and one-third had a BMI ≥ 30 kg/m^2^ (32.7%). Pb and Cd levels increased with age, while the highest level of Hg was found in the 40–59 age group. Non-Hispanic Black participants had the highest levels of Pb and Cd. Levels of Cd, Pb, and Hg decrease with increasing BMI. Pb and Cd levels are higher in smokers (Table 1).

There was a statistically significant negative association between Hg and ln TgAb levels when Hg was analyzed in a continuous manner (β_adjusted_: −0.034 (95% confidence interval (CI): −0.064, −0.005)). No significant associations were found between TgAb levels and Pb and Cd, and anti-TPO and all metals (Table 2).

There was a statistically significant negative association between blood Hg and ln TgAb when comparing Hg quartile 4 to quartile 1 (β_adjusted_: −0.094 (95% CI: −0.185, −0.003)) (Table 3). There was no statistically significant association between Hg levels and ln anti-TPO, nor was there a significant association between Pb and Cd levels and ln TgAb and ln anti-TPO levels (Table 3).

### 3.2. Ecological Study

**Figure 2:**
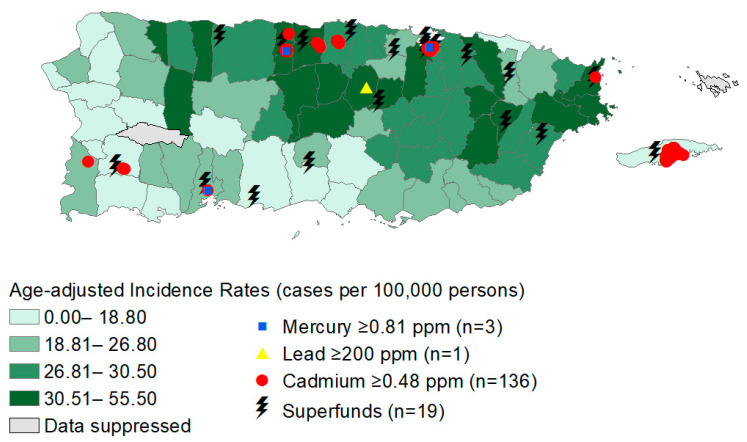
The distribution of age-adjusted thyroid cancer incidence rates per county and sources of mercury, lead, and cadmium exposure equal to or above the maximum contaminant level.

Age-adjusted thyroid cancer incidence rates from 2015 to 2019 per county ranged from 11.1 cases per 100,000 persons to 55.5 cases per 100,000 persons. There were 19 active Superfund sites. Superfund locations were mostly located on the coast of the Puerto Rico, with most sites located on the northern border. Pb levels ranged from 0.57 ppm to 1740 ppm (Supplemental Figure S1). Cd levels ranged from 0 ppm to 15.4 ppm (Supplemental Figure S2). Hg levels ranged from 0 ppm to 32 ppm (Supplemental Figure S3). Higher thyroid cancer incidence rates and increased metal levels in the soil can be observed in the northern region, along with an increased number of Superfund sites (Figure 2).

## 4. Discussion

This study on heavy metal exposure and thyroid cancer incidence in Puerto Rico is the first to combine a large database analysis and an ecological study to explore the impact of metal exposure and Superfund sites on thyroid cancer. Based on our results, Hg may be negatively associated with thyroid cancer, based on TgAb levels, but conclusions should be drawn carefully as these are merely preliminary indications. Our ecological study, however, highlights that areas with increased thyroid cancer incidence rates seem to overlap with metal-polluted areas. These heterogeneous results highlight the importance of further investigating the impact of metal exposure on thyroid carcinogenesis and its effect on public health measures such as the implementation of Superfund sites.

Hg is an element naturally emitted from the earth’s lithosphere to the atmosphere through erosion and volcanism. Human fossil fuel-related activity, through coal combustion and artisanal gold production, has disrupted the natural flux of Hg and led to increasing concentrations of atmospheric Hg since the 1950s [42]. Consequently, Hg is commonly found in the thyroid tissue of adults [43]. Malandrino et al. reported that the concentration of Hg in the thyroid is higher than in adjacent muscle and fat [44]. The literature on the potential association of Hg on thyroid cancer risk remains inconclusive and offers no definitive conclusion for the negative association we observed between Hg and TgAb. A review on Hg exposure and carcinogenesis published in 2022 included three studies that did not report a relationship between Hg exposure (urinary, dietary, fingerprints) and thyroid cancer [45]. However, another study found Hg mass fraction levels to be 17 times higher in malignant thyroid tissue compared to normal thyroid tissue [46]. Kim et al. documented low-level environmental Hg exposure to be associated with increased thyroid cancer risk in a South Korean population [47]. Additionally, another review compiled three thyroid tissue studies and found higher Hg ppm levels in cancerous cases (0.14, 0.06–0.22, n = 178) compared to normal thyroids (0.08, 0.04–0.11, n = 257) [48]. To our knowledge, none of the literature has found a negative association between cancer incidence and Hg levels, as we observed in the first aim of our study.

From a pathophysiology standpoint, Hg’s role on the thyroid has been well-documented. Hg blocks thyroid hormone production by occupying iodine-binding sites and can inhibit or alter hormone action, leading to downstream effects such as hypothyroidism and thyroid inflammation [49]. Hg can activate the RAS/MAPK/PI3K pathway, an essential cascade pathway involved in regulating cellular processes such as cell growth, proliferation, differentiation, and survival, and is connected to mutations in thyroid cancer [48]. Hg also inhibits PTEN and activates the Akt/CREB pathway [50]. The inhibition of PTEN is seen in thyroid cancer as well [48]. Hg is furthermore linked to oxygen free radical production by activating Nrf2 signaling [51,52]. This mechanism is implicated in the development of papillary thyroid cancer as it may promote the survival and growth of tumor cells [53].

Pb and Cd are established endocrine disruptors [25], but their specific role as a potential risk factor for thyroid cancer remains unclear. Pb has become a common pollutant because of industrial activity. From the early 1900s to the early 2010s, Pb was incorporated in many consumer products like petrol, paint, and toys, but has since been legally phased out because of toxicity [54,55,56]. Pb has been linked to breast cancer and pulmonary cancer, suggesting carcinogenicity [57,58]. Mechanistically, Pb is associated with diminished iodine uptake by the thyroid gland [59]. Studies on its effect on TSH, T3, and T4 levels remain inconclusive [60,61,62]. It also causes oxidative stress, which is a mechanism that is associated with metal toxicity and carcinogenicity [63]. However, human studies investigating Pb’s role in thyroid cancer have not established a conclusive association. Li et al. found significantly higher serum lead levels in thyroid adenoma patients than in papillary thyroid cancer patients and nodular goiter patients, implying that Pb plays a role in the development of thyroid diseases [64]. Chung et al. analyzed the blood and thyroid tissue levels of different heavy metals in Korean women undergoing thyroidectomy and found no association between lead and thyroid cancer stage and multifocality [65].

Similar to Hg and Pb, Cd has become a ubiquitous pollutant because of industrial, agricultural, and other human-related processes. While Cd mainly deposits in the liver, kidneys, and muscles, it has also been found in the thyroid gland [66]. Cd binds to cysteine-rich proteins called metallothioneins, which facilitates Cd’s long half-life in the body, anywhere between 5 and 30 years [67]. Past studies in rodents suggest that Cd interferes with TSH, T3, and T4 release as well as disturbs the architecture of thyroid follicular cells in rodents [66,68]. In vitro studies on lymphocytes suggest that Cd exposure alters mitochondrial function and increases Fas death receptor activation as well [69,70]. A combination of these effects may result in altered thyroid function, increasing the risk for the development of thyroid cancer. In alignment with laboratory studies, many retrospective studies have found associations between Cd and thyroid hormone levels [65,71,72]. A previous study found urinary Cd has a positive correlation with thyroid hormones (T3,T4) and thyroid globulin [71]. Similar to our findings, this study did not observe an association between blood Cd and TgAb. In contrast, Ramadan et al. investigated the effect of occupational Cd exposure on workers and found anti-TPO levels were associated with blood Cd levels [72], suggesting that Cd has varied effects on thyroid protein and immunoglobulin levels. The literature available on the relationship between Cd and thyroid cancer is limited. Chung et al. found Cd to be correlated with more advanced thyroid cancer, which aligns with the relationship between the higher incidence of thyroid cancer rates and higher Cd levels in soil we appreciated in the ecological arm of our study [65].

Soil and water sampling in Puerto Rico is currently limited. In July 2018, the EPA published the Metals from Natural and Anthropogenic Sources in Puerto Rico Soils report [23]. At the time, Puerto Rico was home to over 50 contaminated sites under the Superfund and the Resource Conversation and Recovery Act program. Soil samples were taken from 19/50 of the CERCLA/RCRA sites on the island from over 301 locations [23]. To our knowledge, no further soil sampling project of this magnitude has taken place in Puerto Rico since then. Regarding water sampling, the EPA sampled drinking water wells and the Guayama Drinking Water Treatment Plant in southern Puerto Rico between May and September 2023 because of community concerns about coal ash. Results returned with no heavy metal measurements that exceeded limits [73]. Both water and sampling studies were conducted in the past decade, but more robust systems are needed to effectively capture up-to-date soil and water samples from all regions in Puerto Rico and better understand the public health impact.

This study has several limitations. Since this is a cross-sectional study, it is not possible to determine a temporal association between Hg, Pb, and Cd and thyroid-related antibodies. Furthermore, the NHANES study does not record certain variables associated with cancer risk, such as a family history of thyroid cancer and history of genetic mutations associated with increased thyroid risk. Thus, we could not adjust for these factors. Another limitation of the study is that it focuses on the effect of individual metals on thyroid cancer instead of examining mixtures of metal ions on thyroid cancer, which is a more accurate reflection of environmental exposure. Limited studies have examined the relationship between mixtures of metals and thyroid cancer, possibly because of the complexity of synergistic and antagonistic relationships among the metals [25]. Duan et al. found that a blood metal ion mixture of Pb, Cd, and Hg and a urine metal ion mixture containing ten different metal ions were associated with increased cancer mortality (Risk Ratio (RR): 1.41 (95% CI: 1.12, 1.76)) and (RR: 1.60 (95% CI: 1.02, 2.52)), respectively [74]. Additionally, in the ecological study, the soil Hg, Pb, and Cd levels were not available for all regions in Puerto Rico, and samples were only collected from 36% of CERCLA sites on the island [23]. Few measurements of heavy metal levels have been conducted in regions of Puerto Rico with low thyroid cancer incidence rates. We mainly contribute the discrepancy in our results of Hg and thyroid cancer incidence to this lack of data. Consequently, performing a formal analysis to assess the correlation between sampling data and thyroid cancer incidence rates was not feasible and no statistically significant conclusions can be drawn about whether Hg, Mg, and Cd are thyroid cancer predictors.

Our study is the first to investigate the association between metal exposure and thyroid cancer using a large database analysis and an ecological design in Puerto Rico, a unique geographical location, as it is a previous volcanic region. Volcanic areas, as aforementioned, have higher levels of heavy metals due to the nature of eruptions. Additionally, this study includes a nationally representative, population-based sample, so that we were able to account for many potential confounders, such as demographic characteristics, the use of thyroid medication, the existence of thyroid cancer/disease, and pregnancy.

## 5. Conclusions

Although no definitive conclusion on the impact of metal exposure and exposure to Superfund sites on thyroid carcinogenesis can be drawn from the current study, higher thyroid cancer rates in areas with high environmental contamination due to Superfunds warrant future prospective, longitudinal studies in these highly contaminated areas. Due to its geographic location, Puerto Rico would be the ideal setting for such cohort studies.

## Figures and Tables

**Table 1:** A description of the National Health and Nutrition Examination Survey (NHANES) study population (n = 7688).

Variables	Total Population	Lead (μg/dL)	Cadmium (μg/L)	Mercury (μg/L)
	n (%)	GM (SE)	GM (SE)	GM (SE)
**Sex**				
Male	4098 (52.9)	1.479 (0.025)	0.328 (0.007)	1.008 (0.042)
Female	3590 (47.1)	1.048 (0.017)	0.391 (0.008)	0.909 (0.034)
**Age (years)**				
<40	2665 (38.6)	0.931 (0.016)	0.302 (0.007)	0.819 (0.038)
40–59	2552 (39.4)	1.406 (0.025)	0.389 (0.009)	1.079 (0.047)
≥60	2471 (21.9)	1.747 (0.034)	0.406 (0.008)	1.029 (0.049)
**Race/Ethnicity**				
Non-Hispanic White	3422 (67.8)	1.250 (0.024)	0.352 (0.007)	0.912 (0.039)
Non-Hispanic Black	1592 (11.2)	1.303 (0.038)	0.406 (0.009)	0.988 (0.045)
Other	2674 (20.9)	1.258 (0.034)	0.344 (0.009)	1.115 (0.067)
**Body Mass Index (kg/m^2^)**				
<25	2369 (33.2)	1.292 (0.032)	0.403 (0.013)	1.023 (0.049)
25–30	2613 (34.1)	1.325 (0.030)	0.350 (0.012)	1.018 (0.043)
≥30	2706 (32.7)	1.158 (0.021)	0.319 (0.005)	0.846 (0.031)
**Smoking Status**				
Non-smoker	4418 (62.3)	1.158 (0.019)	0.255 (0.004)	0.969 (0.035)
Smoker	3270 (37.7)	1.440 (0.029)	0.617 (0.016)	0.944 (0.045)

Abbreviations: GM: Geometric Mean; SE: Standard Error.

**Table 2:** Association between continuous lead, cadmium, and mercury levels and thyroid antibodies.

	Ln TgAb (IU/mL)	Ln anti-TPO (IU/mL)
	B_adj_ * (95% CI)	B_adj_ * (95% CI)
Ln Lead	0.034 (−0.022, 0.089)	0.074 (−0.014, 0.162)
Ln Cadmium	−0.014 (−0.063, 0.036)	0.0003 (−0.071, 0.072)
Ln Mercury	**−0.034 (−0.064, −0.005)**	0.006 (−0.049, 0.060)

* Adjusted for age group, sex, race/ethnicity, BMI group, iodine status, smoking status. Bolded font indicates associations where the *p*-value is <0.05. Abbreviations: TgAb: thyroglobulin antibodies; TPO: thyroid peroxidase; CI: confidence interval.

**Table 3:** Association between lead, cadmium, and mercury levels in quartiles and thyroid antibodies.

	Ln TgAb (IU/mL)	Ln Anti-TPO (IU/mL)
	B_adj_ * (95% CI)	B_adj_ * (95% CI)
**Ln Lead**		
Quartile 1	Ref	Ref
Quartile 2	0.005 (−0.072, 0.082)	0.006 (−0.122, 0.134)
Quartile 3	0.006 (−0.101, 0.114)	0.008 (−0.136, 0.151)
Quartile 4	0.027 (−0.081, 0.136)	0.100 (−0.054, 0.253)
**Ln Cadmium**		
Quartile 1	Ref	Ref
Quartile 2	0.040 (−0.067, 0.147)	0.140 (−0.061, 0.341)
Quartile 3	0.018 (−0.088, 0.123)	0.114 (−0.040, 0.268)
Quartile 4	0.013 (−0.100, 0.126)	0.110 (−0.090, 0.310)
**Ln Mercury**		
Quartile 1	Ref	Ref
Quartile 2	−0.052 (−0.147, 0.042)	−0.065 (−0.210, 0.080)
Quartile 3	−0.051 (−0.135, 0.033)	0.005 (−0.169, 0.180)
Quartile 4	**−0.094 (−0.185, −0.003)**	−0.050 (−0.206, 0.107)

* Adjusted for age group, sex, race/ethnicity, BMI group, iodine status, smoking status. Bolded font indicates associations where the *p*-value is <0.05. Abbreviations: TgAb: thyroglobulin antibodies; TPO: thyroid peroxidase; CI: confidence interval.

## Data Availability

No new data were created or analyzed in this study. Data sharing is not applicable to this article.

## Supplementary Materials

The following supporting information can be downloaded at https://www.mdpi.com/article/10.3390/toxics12090632/s1, Figure S1: lead levels and thyroid cancer incidence rate in Puerto Rico; Figure S2: cadmium levels and thyroid cancer incidence rate in Puerto Rico; Figure S3: mercury levels and thyroid cancer incidence rate in Puerto Rico.
