# Supplementary figures and images for: The Association between Metals and Thyroid Cancer in Puerto Rico—A National Health and Nutrition Examination Survey Analysis and Ecological Study
Source: Toxics. 2024 Aug 28;12(9):632. doi: 10.3390/toxics12090632 (PMC11435839; doi:10.3390/toxics12090632)

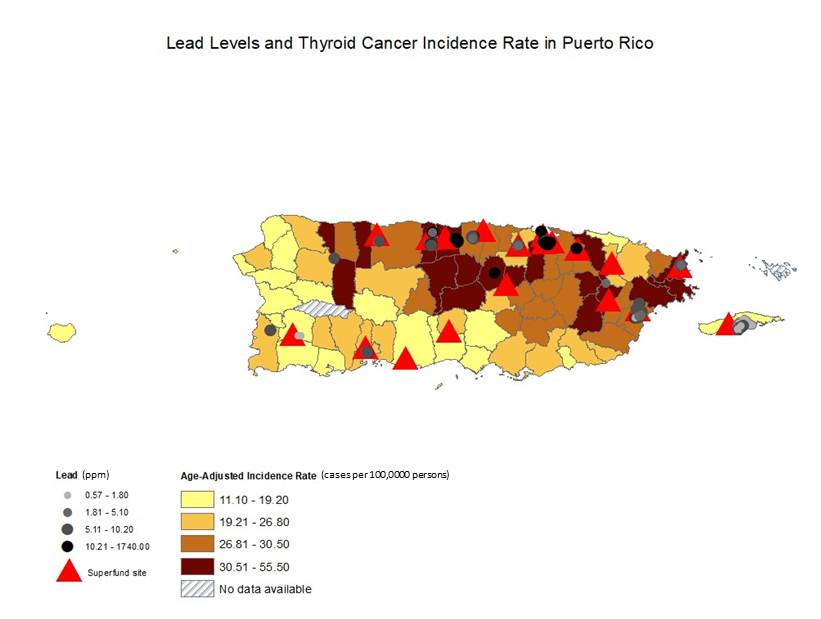

Supplement: Supplementary file 1 [file toxics-12-00632-s001.zip › Supplementary Figure S1.tif]

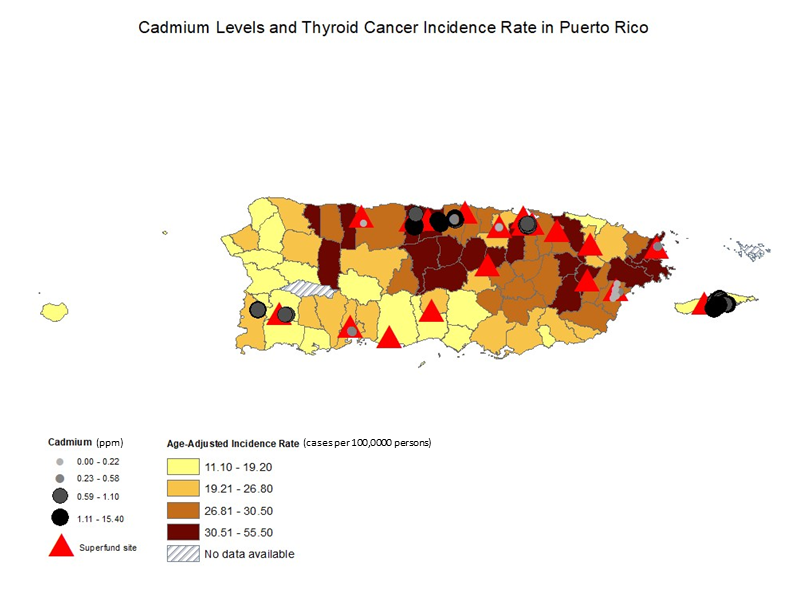

Supplement: Supplementary file 1 [file toxics-12-00632-s001.zip › Supplementary Figure S2.tif]

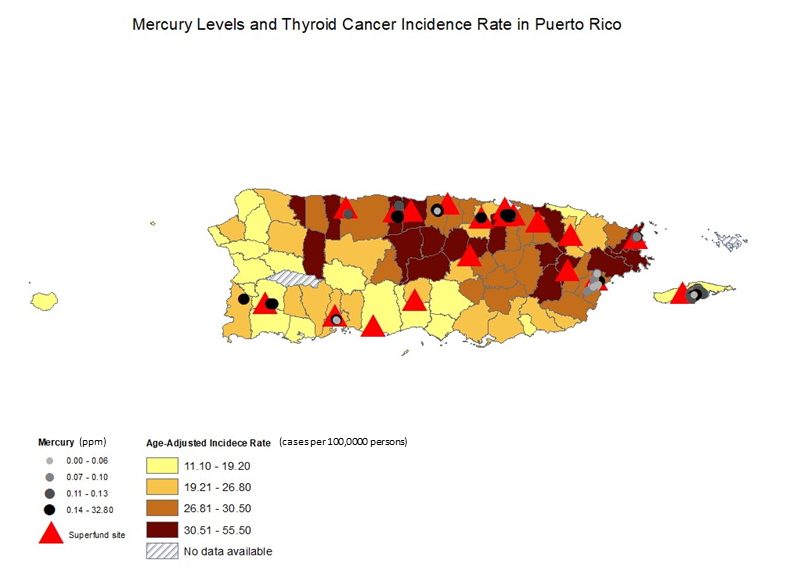

Supplement: Supplementary file 1 [file toxics-12-00632-s001.zip › Supplementary Figure S3.tif]
